# Fermentation of Microalgae as a Platform for Naturally Encapsulated Oil Powders: Characterization of a High-Oleic Algal Powder Ingredient

**DOI:** 10.3390/microorganisms13071659

**Published:** 2025-07-14

**Authors:** Walter Rakitsky, Leon Parker, Kevin Ward, Thomas Pilarski, James Price, Mona Correa, Roberta Miller, Veronica Benites, Dino Athanasiadis, Bryce Doherty, Lucy Edy, Jon Wittenberg, Gener Eliares, Daniel Gates, Manuel Oliveira, Frédéric Destaillats, Scott Franklin

**Affiliations:** Checkerspot, Inc., Alameda, CA 94501, USA

**Keywords:** algae fermentation, encapsulation-free oil powder, oil delivery system, oil powder, lipid rich powder

## Abstract

Powdered oil ingredients are widely used across food, nutrition, and personal care industries, but they are typically produced through encapsulation technologies that involve multiple additives and stabilizers. These systems can compromise oxidative stability, clean-label compliance, and functional performance. Here, we present the development and characterization of a novel high-oleic algal powder (HOAP) produced from a heterotrophically fermented microalgae. The production strain was developed through classical mutagenesis to enhance oleic acid and lipid accumulation. Three independent fermentation batches at a 20 L scale demonstrated strong reproducibility in key metrics, including dried-cell weight (210.0 g per L on average, CV% = 0.7), oil content (62.0% of DCW on average, CV% = 2.0), and oleic acid (88.8% of total fatty acids on average, CV% = 0.1). HOAP exhibited a favorable nutritional profile (e.g., high monounsaturated fat and fiber, low sugar and moisture) and good oxidative stability under ambient and accelerated storage conditions. Microbiological analyses confirmed compliance with food-grade standards, and in silico allergenicity screening revealed no clinically relevant homologs. Unlike traditional oil powders, HOAP does not require encapsulation and retains oil within a natural protein–fiber matrix, offering both functional and clean-labeling advantages. Its compositional attributes and stability profile support potential use in food, nutrition, and the delivery of bioactive nutrients. These findings establish HOAP as a next generation of oil powder ingredients with broad application potential.

## 1. Introduction

Powdered oil ingredients are widely used across the food, nutrition, and personal care industries as convenient, shelf-stable alternatives to liquid oils [1]. In food and beverage applications, oil powders enable the inclusion of lipids in dry products, such as powdered soups, instant sauces, baking mixes, and protein shakes, where the presence of liquid oil would be technically incompatible or logistically impractical. In specialized nutrition, including sports nutrition, medical foods, and infant formula, powdered oils allow for accurate dosage, ease of transport, and the extended shelf life of sensitive nutrients [1,2,3]. In all these applications, oxidative stability, lipid load, solubility, and label acceptability are critical parameters.

Despite their broad utility, most commercial oil powders are produced via encapsulation technologies, typically by spray drying emulsified oil blends with carrier matrices composed of maltodextrins, modified starches, milk proteins, or gums [1,4,5]. While effective in generating oil-in-powder formats, these systems generally incorporate multiple additives, such as emulsifiers, stabilizers, and sometimes preservatives, that limit clean-label potential. Moreover, encapsulation yields a lipid distribution where the oil is physically entrapped rather than structurally integrated, making such powders prone to phase separation, oil leakage, cacking, and oxidative degradation over time. These constraints are particularly challenging in applications requiring high fat load, additive-free formulation, or stability under elevated temperatures and humidity.

To address these limitations, we developed high-oleic algal powder (HOAP), a lipid-rich powder derived directly from the biomass of a heterotrophically grown microalga. HOAP is not an encapsulated system, but a whole cell with intrinsically high lipid content (>60%), particularly enriched in monounsaturated oleic acid (~89% of total fatty acids). This configuration allows the oil to be stably integrated within a protein–fiber–lipid matrix, providing structural integrity of the powder.

Importantly, HOAP also presents unique potential as a naturally structured delivery system for lipophilic compounds and bioactive fatty acids. Its high oil content, combined with the presence of emulsifying cell wall and membrane components in a fibrous matrix, may enhance the dispersibility and bioavailability of incorporated molecules in nutritional products. This could be advantageous for applications seeking to deliver functional lipids (e.g., omega-3 or fat-soluble vitamins) or other hydrophobic nutraceuticals within stable, powder-based systems. Unlike synthetic encapsulates, HOAP’s native biological matrix offers a simplified and potentially more bioavailable/digestible vehicle for lipid-associated bioactives.

The strain used for HOAP production has been previously documented and has been developed through classical mutagenesis and adaptive selection for enhanced lipid accumulation and oleic acid content [6]. This strain has previously been used to produce a high-quality refined oil extensively characterized for use in food, personal care, and industrial formulations [6]. Building on this foundation, the current work describes the production process and physicochemical characterization of HOAP and evaluates its batch-to-batch reproducibility, compositional profile, microbiological and allergenic safety, and storage stability. HOAP offers a differentiated solution for formulators seeking additive-free, high-fat, shelf-stable ingredients with clean-label appeal. This manuscript aims to provide a comprehensive technical and safety profile of HOAP and to support its potential use as a next-generation lipid structuring ingredient for a wide range of applications in food, nutrition, and beyond.

## 2. Materials and Methods

**Strain development.** Classical strain improvement was initiated on wild-type *Prototheca moriformis* strain isolate UTEX 1533 displaying levels of 28% and 56–60% of palmitic and oleic acids, respectively, as previously described [6]. Briefly, classical strain improvement regimes included repeated rounds of UV (8000–20,000 μJoules) and chemical (270 mM ethyl methane sulfonate (EMS), 45 min @ 32 °C; 4-nitroquinoline 1-oxide-4-NQO, at concentrations, exposure times, and temperatures ranging from 2.7–60 μM, 5–30 min, and 28–32 °C, respectively) mutagenesis. Enrichment strategies performed after mutagenesis included both chemical (cerulenin, a beta-keto-acyl-ACP synthase inhibitor at 7–50 μM; AZD-8055 an mTOR kinase inhibitor at 26–75 μM and clomiphene, an inhibitor of sterol biosynthesis at 12–100 μM) and physical (buoyant density centrifugation) means. The iterative methodology involved the creation of mutant libraries followed by clonal isolation, assessments of feedstock utilization and growth, lipid accumulation, and the validation of oil composition. Screening these libraries led to the identification of isolates with the ability to produce elevated levels of oleic acid, aligning with the targets for high-oleic acid substitutes. Detailed conditions for the production of the high-oleic algae strain are provided elsewhere [6,7].

**Culture media composition.** Cultured conditions and media used has been described in a previous paper [6,7]. Briefly, vegetative growth medium was comprised of macronutrients including NaH_2_PO_4_, K_2_HPO_4_, citric acid monohydrate, magnesium sulfate heptahydrate, calcium chloride dihydrate, and dextrose at 1.64, 1.98, 1.05, 1.23, 0.02, and 40 g/L, respectively. Ammonium sulfate served as the sole nitrogen source at 1.0 g/L. Anti-foam (Sigma 204, Sigma-Aldrich, St Louis, MO, USA) was added to a final concentration of 0.225 mL/L. Trace minerals were prepared as a 1000 × stock solution comprised of boric acid, zinc sulfate heptahydrate, manganese sulfate monohydrate, sodium molybdate dihydrate, nickel nitrate heptahydrate, citric acid monohydrate, copper (II) sulfate pentahydrate, and iron (II) sulfate heptahydrate at 0.91, 1.76, 1.23, 0.05, 0.04, 20.49, 0.05, and 0.75 g/L, respectively. A 1000 × vitamin stock was comprised of thiamine HCl, D-pantothenic acid hemicalcium salt, biotin, cyanocobalamin, riboflavin, and pyridoxine HCl at 3.0, 0.16, 0.0048, 0.00034, 0.015, and 0.0078 g/L, respectively. Lipid production medium was identical to vegetative growth medium except that ammonium sulfate was supplied at 0.2 g/L. All solutions were filter sterilized prior to use.

**Production process.** The production process was performed at the 20 L reactor scale essentially as described previously [6,7]. The seed train for each run was initiated from a single cryovial from a master cell bank of the HOAP strain, which was used to inoculate 50 mL of lipid production medium in a 250 mL baffle flask grown at 28 °C with shaking (200 rpm). This culture was transferred once it reached an A750 of 10–15 to two, 1 L baffle flasks, each containing 200 mL of lipid production medium. These flasks were grown with shaking (200 rpm) at 28 °C until they also reached an A750 of 10–15, at which point their contents were sterilely transferred to a 20 L fermentation vessel, supplying a 10% inoculum (20 L M-Series, Solaris Biotech, Porto Mantovano (MN) Italy. Fermentation conditions included an inoculation volume of 0.25–0.3 of the fermenter volume, with pH and dissolved oxygen (DO) setpoints of 5.5–6.0 and 30%, respectively. The operating temperature was 28 °C throughout with aeration and agitation managed to maintain DO. Fermentation medium was fortified to support higher cell density as follows. Medium composition remained the same; however, trace metals and vitamin solutions were increased 15 and 10-fold, respectively. Macronutrients (sodium phosphate, potassium phosphate, citric acid monohydrate, magnesium sulfate heptahydrate, and calcium chloride dihydrate) were increased to 7.13, 9.25, 2.1, 17.33, and 0.8 g/L, respectively. Glucose feed consisted of a 55% wt.: wt. sterile solution. At the conclusion of the fermentation, the resulting fermentation broth was pasteurized at 65 °C for 30 min, prior to being dried on a double drum dryer (Buflovak Model ADDD operating at 70 psig steam, 1200 rpm; Buflovak, Tonawanda, NY, USA) such that the final moisture content was <2%.

**Analysis of the high-oleic algal oil powder (HOAP).** For all analytes measured, the average, standard deviation (SD), and coefficient of variation (CV%) were calculated based on HOAP samples recovered from three non-consecutive production batches. This approach was used to assess the reproducibility and robustness of the production process. The compositional profile of HOAP powder (HOAP) was assessed using standardized and validated analytical methods. Visual appearance was evaluated by macroscopic inspection for consistency in color and texture. Moisture content was determined using an internal gravimetric method based on Standard NF EN ISO 662, and ash content was measured via incineration using an internal method aligned with Standard ISO 6884:2008. Protein content was quantified using the Kjeldahl method (Standard NF V 04-407), while total fat content was extracted and analyzed using the Folch method [8], which relies on a chloroform–methanol extraction system. Sucrose concentration was determined using an enzymatic colorimetric method. Dietary fiber content was measured following the Standard AOAC Official Method 985.29, which includes enzymatic digestion and the gravimetric determination of both soluble and insoluble fiber fractions. Amino acid analysis was conducted using the following two complementary methods: tryptophan content was quantified in accordance with Standard EU Regulation 152/2009, while all other amino acids were analyzed using the Standard ISO 13903:2005 protocol. Biogenic amines, including histamine, tyramine, putrescine, cadaverine, and spermidine, were quantified using high-performance liquid chromatography (HPLC) with pre-column derivatization, following the procedure described elsewhere [9]. This method involves the dansyl chloride derivatization of amines and HPLC separation using a C18 column, with detection by UV absorbance at 254 nm. All compositional analyses were performed on three independent production batches of HOAP to assess batch-to-batch variability and ensure data robustness.

**Fatty acid (FA) analysis.** Fatty acid composition of algae and high-oleic sunflower oil samples were measured as their fatty acid methyl esters (FAMEs) following direct trans-esterification with a sulfuric acid methanol solution [6]. Samples were injected on an Agilent 8890 gas chromatograph system equipped with a split/split less inlet and a flame ionization detector (Agilent Technologies, Palo Alto, CA, USA). An Agilent DB-WAX column (30 m × 0.32 mm × 0.25 um dimensions) was used for the chromatographic separation of the FAME peaks. A FAME standard mixture purchased from Nu-Chek Prep (Nu-Chek Prep Inc., Elysian, MN, USA) was injected to establish retention times. Response factor corrections were previously determined empirically using various standard mixtures from Nu-Chek Prep. Methyl nonadecanoate (19:0) was used as an internal standard for the quantitation of individual FAMEs. Run-to-run reproducibility was controlled by running an internal reference standard, an algal biomass control sample. As an example, this standard, run over three years and assessing 18 random runs, shows a standard deviation in oil content (g/L) of 0.80 g/L (average of 49.14), oleic acid content of 0.13 area % (average of 83.60), total saturate content of 0.05 area % (average of 7.43), total monounsaturated FA content of 0.14 area % (average of 84.68), and total polyunsaturated FA content of 0.03 area % (average of 7.51). The coefficient of variation (CV%) across these five parameters was 1.62, 0.15, 0.73, 0.17, and 0.40 percent, respectively. Such low analytical variability allows us to pick-up small variations of targeted fatty acid levels when performing the high-throughput screening of classically improved strains.

**Microbiological analysis.** The microbiological quality and safety of HOAP were assessed using accredited standard methods. Total aerobic plate counts were determined in accordance with Standard NFEN ISO 4833-2, while coliform counts were evaluated using Standard NF V08-050. *Escherichia coli* (*E. coli*) detection was performed following the Standard NF ISO 16649-2 protocol. The presence of *Salmonella* spp. was assessed in 25 g of samples using the method outlined in Standard BKR 23/07-10/11. The enumeration of coagulase-positive *Staphylococci* was conducted using Standard NF EN ISO 6888-2, and Pseudomonas spp. were quantified using Standard ISO 22717. Fungal contamination was evaluated by separate determination of mold and yeast counts, both performed according to Standard NF V08-036. All microbial analyses were conducted in triplicate on three independent production batches to evaluate compliance with quality standards and to confirm microbiological safety of the final product.

**In silico assessment of allergenicity.** An in silico assessment of potential allergenicity was performed using the predicted amino acid sequences from the *P. moriformis* base strain. Protein sequences were derived from an in-house genome assembly generated using PacBio HiFi sequencing, assembled with Canu [10], scaffolded with RagOut [11], and annotated using Augustus [12] with a model trained on *Chlamydomonas reinhardtii* and transcriptome data from *Auxenochlorella protothecoides*. The resulting amino acid sequences were compared against the COMPARE allergen database [13] using BLASTp (NIH, Bethesda, MD, USA) with an E-value threshold of 1 × 10^−5^ (Supplementary Material Table S1 and Figure S1).

## 3. Results

### 3.1. Strain Development

**Figure 1:**
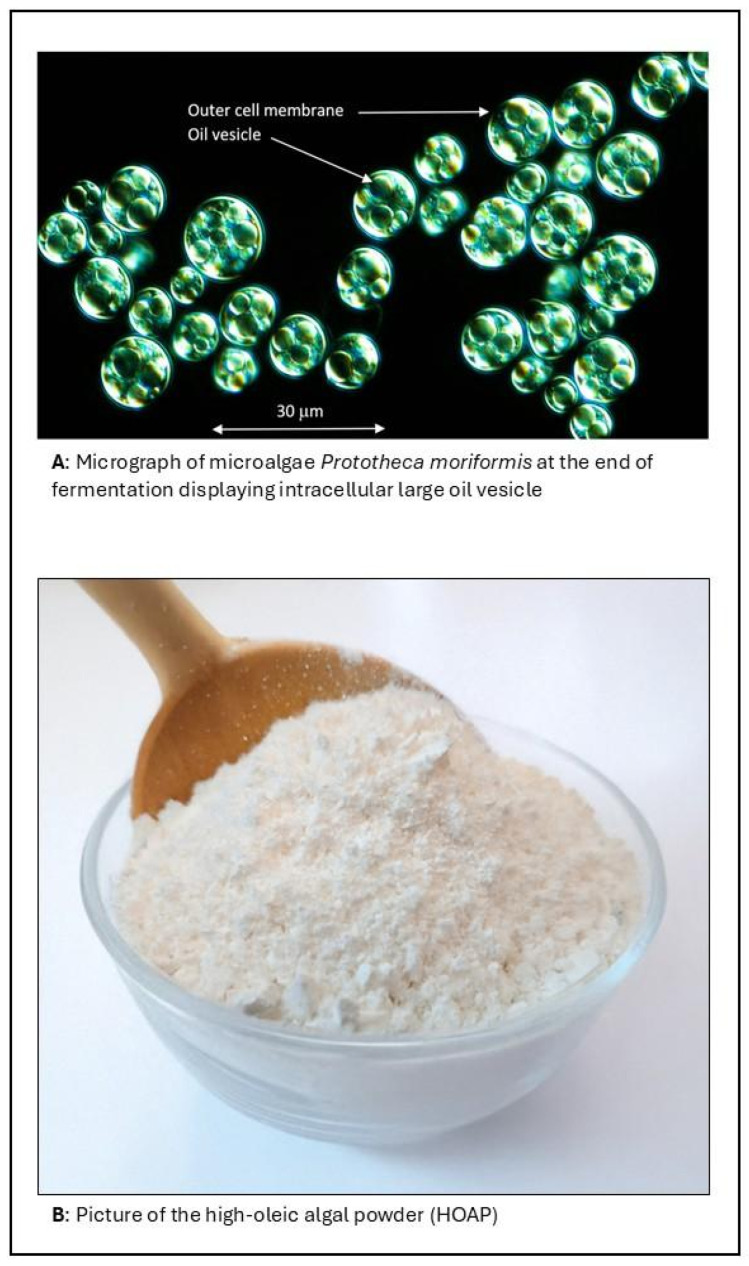
(**A**) Micrograph of *Prototheca moriformis* cells at the end of fermentation. The image reveals numerous large oil vesicles within the cells, confirming high lipid accumulation. The outer cell membrane and oil vesicles are indicated. Scale bar: 30 µm. (**B**) Photograph of the resulting high-oleic algal powder (HOAP) obtained after fermentation, thermal inactivation, and drying. The powder is pale yellow-colored and has a bulk density of 0.63 kg per L.

The initial production strain was derived from UTEX 1533, originally deposited by W.B. Cooke in 1966 at the University of Texas Culture Collection of Algae (UTEX, Austin, TX, USA) and annotated as *Prototheca wickerhamii*. Sequence analysis of the 23S ribosomal DNA (rDNA), however, confirmed that UTEX 1533 is taxonomically consistent with other *P. moriformis* isolates, as previously described [14], showing 100% identity with the 23S rDNA sequence of ATCC 16529. The *P. moriformis* base strain is a unicellular alga with a typical cell diameter of 10–12 μm (Figure 1A) and was cultured under standard conditions (28 °C, pH~6.0) for further development. To enhance lipid productivity and specifically increase oleic acid content, the base strain was subjected to classical mutagenesis using both chemical and physical methods, as described previously by Parker and co-workers [6]. Mutants exhibiting enhanced glucose utilization and oleate production were selected and stabilized through multiple rounds of sub-culturing, leading to the isolation of intermediate strains. These strains underwent further improvement through ethyl methane sulfonate (EMS) mutagenesis combined with selection in lipid-inducing media containing membrane fluidizing agents. These subsequent strains were subjected to additional rounds of chemical and UV mutagenesis and selection based on glucose consumption and fatty acid profile. The final selected strain exhibited a significantly enhanced oleic acid content compared to the original base strain and was selected as the production strain for HOAP.

### 3.2. Production Process

The seed train for HOAP and its production were described above under Materials and Methods. Throughout the process, factors impacting fermentation, including the pH, temperature, agitation, and aeration rates, were controlled. At the conclusion of the fermentation, the fermentation broth was pasteurized, as described in Materials and Methods. The fermentation broth was dried for final water removal (<2% residual moisture). The HOAP is a fine powder (bulk density = 0.63 kg/L) with a uniform light beige to pale yellow color and a neutral odor (Figure 1B).

To evaluate the reproducibility of the HOAP production process, three independent fermentation runs were conducted at 20 L scale under identical conditions. The fermentations were performed in glucose-based heterotrophic media with controlled temperature (~28–30 °C), pH (~6.0), and aeration, as described above. Key fermentation metrics, including oleic acid percentage, dry cell weight (DCW), oil content, and oil titer, were monitored to assess batch-to-batch variability. The results demonstrated a high level of consistency across all runs (Table 1). Oleic acid content averaged 88.9% of total fatty acids, with a coefficient of variation (CV) of only 0.1%, confirming excellent reproducibility in fatty acid composition. DCW values ranged from 208.0 to 212.0 g/kg, averaging 210.0 g/kg, with a CV of 1.0%, indicating stable biomass production. Oil content, expressed as a percentage of DCW, remained consistently high across all batches, with an average of 62.0% and a low CV of 2.8%. Oil titers in fermentation broth at the conclusion of the run ranged from 128.1 to 133.1 g/L, yielding an average of 130.2 g/L (CV = 2.0%), further supporting the robustness of the process in achieving high lipid productivity. These data confirm the strong reproducibility of the fermentation process for HOAP at bench scale, particularly in the production of a high-oleic lipid profile and oil content of the resulting biomass. As reported in our previous study [6], the production of HOAP has already been successfully scaled up to the 400 L level, providing a solid foundation for further industrial-scale development. The low variation across multiple performance metrics reinforces the reliability of both the high-oleic algae strain developed and the fermentation protocol, supporting its suitability for industrial scale-up and commercial applications.

### 3.3. Nutritional Composition

To assess the robustness and reproducibility of the HOAP production process, three non-consecutive production batches were performed under identical conditions. To assess batch-to-batch consistency, the average, standard deviation (SD), and coefficient of variation (CV%) were calculated for all analytes measured across the three production batches. This statistical treatment provides a clear evaluation of the reproducibility of the compositional profile and confirms the reliability of the process.

The nutritional profile of HOAP is characterized by a high lipid content and a significant amount of dietary fiber, with consistently low levels of moisture and ash. Across three production batches, the average fat content was 64.5 g/100 g (CV = 1.9%), confirming the lipid-rich nature of the material. Fiber content averaged 34.4 g/100 g (CV = 9.0%), contributing to the bulk of the non-lipid fraction, while protein content was modest at 3.2 g/100 g (CV = 3.1%). The sucrose content was low (average 1.3 g/100 g, CV = 7.7%), and the residual moisture averaged 2.4 g/100 g (CV = 28.9%), reflecting an effective drying process (Table 2).

Fatty acid analysis confirmed that the lipid fraction is predominantly composed of monounsaturated fatty acids (MUFA), with oleic acid (18:1 n–9) representing an average of 88.9% (Table 1 and Table 3) of total fatty acids. Saturated fatty acids (SFA) were present in smaller proportions, including palmitic acid (16:0; 3.7%, CV = 1.5%) and stearic acid (18:0; 1.7%, CV = 4.7%). Polyunsaturated fatty acids (PUFA) were also detected at low levels, including linoleic acid (18:2 n–6; 3.3%, CV = 4.5%) and α-linolenic acid (18:3 n–3; 0.2%, CV = 2.4%). This fatty acid profile aligns with that of conventional high- and ultra-high oleic oils, indicating suitability for use in applications where oxidative stability and a favorable fatty acid profile are desired.

The amino acid composition revealed the presence of all essential amino acids in varying amounts, with leucine (0.26 g/100 g, CV = 4.38%), lysine (0.17 g/100 g, CV = 5.88%), and valine (0.20 g/100 g, CV = 5.87%) among the most abundant. Non-essential amino acids, such as glutamic acid (0.33 g/100 g, CV = 9.84%), alanine (0.27 g/100 g), and aspartic acid (0.26 g/100 g, CV = 4.33%) were also present in appreciable levels. Methionine and ornithine were detected at low or trace levels (<0.05 g/100 g), while hydroxyproline was below the quantification threshold in all batches (Table 4). These data support the presence of a well-rounded and limited protein component in HOAP, consistent with its primary use as a fat-rich structuring ingredient rather than a protein source.

Biogenic amine analysis of HOAP samples from three production batches confirmed the absence of detectable levels of cadaverine, histamine, phenylethylamine, putrescine, spermine, tryptamine, and tyramine, based on the limits of detection of the analytical methods employed. Spermidine was the only biogenic amine consistently detected across all batches, with an average concentration of 74.8 mg/kg (range: 68.0–79.5 mg/kg, CV = 8.0%). These findings indicate that HOAP is largely free of biogenic amines, aside from low levels of spermidine (Table 5), the latter of which may be high due to spermidine’s role in coping with abiotic stress, of which such high levels of lipid production would most definitely qualify [15,16,17,18,19].

### 3.4. Stability of HOAP

A two-month stability study was conducted on HOAP stored at 23 °C and 45 °C under vacuum-sealed conditions, with and without the addition of the antioxidant butylated hydroxytoluene (BHT) at concentrations of 0, 20, and 100 ppm. Peroxide value, fat content, and appearance were monitored over time to assess oxidative and physical stability (Table 6). At 23 °C, samples without antioxidant showed a gradual increase in peroxide value from 0.9 to 2.8 meq O_2_/kg over two months. In contrast, samples containing BHT remained more stable, with peroxide values decreasing slightly or fluctuating minimally (from 0.5 to 0.6 meq O_2_/kg for 100 ppm BHT and from 0.8 to 0.5 meq O_2_/kg for 20 ppm BHT). At 45 °C, the peroxide value increased more markedly in the absence of antioxidant, rising from 0.9 to 3.5 meq O_2_/kg by month one and remaining stable thereafter. Samples with added BHT showed moderate increases (from 0.5 to 2.3 meq O_2_/kg for 100 ppm and from 0.8 to 2.3 meq O_2_/kg for 20 ppm), but peroxide values remained below 5 meq O_2_/kg in all conditions. Fat content remained relatively stable across all conditions, with slight fluctuations observed over the two-month period. No consistent trend in fat degradation or concentration was detected, and final fat content values ranged from 59.17 to 61.21 g/100 g across all samples. Physical appearance was unchanged in all samples regardless of temperature or antioxidant content, with all batches conforming to the original visual specifications throughout the test period.

These results demonstrate that HOAP exhibits good oxidative and physical stability when stored at ambient or elevated temperatures for at least two months. The addition of BHT, even at low concentrations, further enhances oxidative stability.

### 3.5. Microbiology

Microbiological analysis was conducted on three production batches of HOAP to assess the product’s microbial safety and compliance with food-grade quality standards (Table 7). All batches exhibited low total aerobic plate counts, ranging from 80 CFU/g to 3000 CFU/g. Coliforms and *Escherichia coli* were not detected above the quantification threshold in any batch (<10 CFU/g), and *Salmonella* was absent in 25 g of sample across all batches. Similarly, no detectable levels of *Pseudomonas aeruginosa* or coagulase-positive *Staphylococci* were found (<10 CFU/g), and mesophilic aerobic spores were also below 10 CFU/g in all cases.

Fungal contaminants, including mold and yeast, were present at levels below 1000 CFU/g in all samples, well within the acceptable limits for powdered food ingredients. No deviations in microbial profile were observed between batches. These data confirm that HOAP meets microbiological criteria expected of shelf-stable, low-moisture food ingredients and suggest that the production process, encompassing fermentation, thermal inactivation, and drying, effectively minimizes microbial risk.

### 3.6. Allergenicity Assessment

An in silico assessment of the allergenicity potential for HOAP has been performed by comparing the digestibility of its proteins and a comparison of amino acid sequences of said proteins with sequences of known allergenic proteins. It assumes that the proteins found in the HOAP are expected to be digested, absorbed, metabolized, and excreted in the same manner as other types of plant-derived proteins in the human diet. A comparison of the predicted amino acid sequences from *P. moriformis* base strain, *Auxenochlorella protothecoides* [20], and *Chlorella variabilis* [21] were compared with known allergenic proteins via the annotated COMPARE allergen database [13]. The inclusion of potential allergens to commonly queried databases like COMPARE generally requires the protein sequence to trigger a positive immunoglobulin E (IgE) binding.

A total of 607 sequence hits exceeding an E-value threshold of 1 × 10^−5^ were identified in the base strain, with the majority (n = 553) shared with both *A. protothecoides*. *Chlorella variabilis* showed the highest number of lineage-specific matches (n = 143), relative to the base strain (n = 5) and *A. protothecoides* (n = 3), consistent with its higher number of predicted protein-coding genes (see Supplementary Figure S1). Filtering for sequences with greater than 70% identity to known allergens yielded six candidate proteins in the base strain (Supplementary Table S1 and Figure S1). These candidates included alpha-tubulin, heat shock protein 70 kDa (Hsp70), glyceraldehyde-3-phosphate dehydrogenase (GAPDH), two cyclophilins, and a casein kinase (CK2). Each of these proteins was further evaluated against IgE epitope databases using AlgPred2 for both direct IgE binding matches and motif similarity. No IgE epitope or motif matches were identified for any of the six candidates.

## 4. Discussion

The development of a HOAP production strain demonstrates the effective application of classical mutagenesis and phenotypic selection to enhance both lipid productivity and oleic acid content. Starting from a well-characterized axenic isolate, sequential rounds of chemical and UV mutagenesis led to the identification of a production strain with markedly improved biosynthetic capacity [6]. The fermentation process, implemented under tightly regulated conditions, exhibited strong reproducibility. Across three independent 20 L-scale fermentations, key metrics, including oleic acid content, dry cell weight (DCW), oil content, and oil titer, showed minimal variation (CVs < 2.0%), confirming the genetic stability of the production strain and the robustness of the production process (Table 1).

A compositional analysis of HOAP confirms its designation as a lipid-rich, fiber-containing material with low moisture and sugar content (Table 2). With an average fat content of 64.5 g/100 g, HOAP lies within the upper range for microbial lipid ingredients and meets the functional requirements for use in food applications demanding high lipid loads (Table 2). The elevated fiber content (34.4 g/100 g) enhances its potential as a dual-function ingredient, delivering both lipid and dietary fiber. Low moisture content (2.4 g/100 g) further contributes to shelf stability and reduces microbial risk, as confirmed by microbial safety data (Table 7). The fatty acid profile, dominated by oleic acid (88.8% of total fatty acids), closely resembles that of high-oleic sunflower or canola oils, conferring excellent oxidative and thermal stability. Low levels of saturated (e.g., palmitic and stearic) and polyunsaturated fatty acids (e.g., linoleic and α-linolenic acids) support both health-oriented and formulation-stable applications. The amino acid composition includes all essential amino acids, most notably leucine, lysine, and valine albeit at low concentrations consistent with its primary role as a structuring fat rather than a protein supplement (Table 4). The detection of spermidine (74.8 mg/kg), a naturally occurring polyamine, provides an additional nutritional benefit (Table 5), present at levels higher to those found in legumes, cheeses, and soybeans. Spermidine is known to support autophagy, lipid metabolism, and cellular homeostasis [17,18,19], and HOAP may serve as a valuable dietary source of this bioactive compound. These compositional characteristics confirm HOAP’s suitability for use in formulations requiring lipid structuring, oxidative stability, and added nutritional functionality. The stability study presented here further reinforces HOAP’s commercial readiness, demonstrating its resistance to oxidation and physical degradation under both ambient and elevated storage conditions. Fat content remained stable over time, and no changes in appearance were observed, suggesting the matrix integrity of the powder was preserved. These findings underscore HOAP’s potential for use in shelf-stable food systems and support its inclusion in formulations requiring resilience to heat, oxygen, and long-term storage (Table 6). The stability profile is comparable to those reported for other high-lipid microbial powders. This confirms the utility of antioxidant strategies for extending product shelf life when needed.

The overall safety profile of HOAP is supported by both allergenicity and microbiological assessments. Given the close phylogenetic relationship between *Prototheca* and *Auxenochlorella*, it may be relevant to reference the published safety study of a *Chlorella*-based whole algal flour in a rat model, which demonstrated no adverse effects at high doses [22], to further support the safety profile of HOAP. In silico analysis of the *P. moriformis* base strain proteome revealed only six proteins with moderate sequence similarity (>70%) to known allergens in the COMPARE database (Table S1). Further screening using AlgPred2 confirmed that none of these candidates possessed IgE-binding epitopes or allergenic motifs. These proteins, including alpha-tubulin and heat shock protein 70 kDa, are commonly identified in BLAST-based screens of microbial genomes and have not been associated with clinically relevant allergic reactions in vivo. Similar findings have been reported for related microalgae, such as *Chlorella variabilis* and *A. protothecoides*, which share a comparable allergen database profile and have established use in food and feed applications. In parallel, the risk of Protothecal infection is considered negligible. Although *Prototheca* species are occasionally implicated in opportunistic infections (protothecosis), such cases are extremely rare and primarily involve *P. wickerhamii* in immunocompromised individuals. The production strain used was derived from a *P. moriformis* isolate classified under Biosafety Level 1 (BSL-1), indicating no known risk to healthy individuals. Furthermore, the manufacturing process includes a validated thermal inactivation step (>65 °C for at least 30 min), ensuring complete microbial deactivation and preventing the presence of any viable cells in the final product. Collectively, the absence of known allergenic proteins with clinical relevance, the BSL-1 classification of the production organism, and the robust thermal processing step confirm that HOAP presents no significant safety concerns for human consumption.

The unique compositional and functional attributes of HOAP position it as a promising ingredient for applications in the food and nutrition space. Unlike conventional oil powders, which are typically produced through the encapsulation of liquid oils with carrier agents, such as maltodextrins or proteins [1,4,5], HOAP is an intrinsically structured lipid-rich biomass that does not require encapsulation to achieve a dry powder format (Figure 1B). This provides significant advantages in terms of ingredient simplicity, clean labeling, and process efficiency. The high content of oleic acid confers excellent oxidative stability and a favorable fatty acid profile aligned with current nutritional guidelines promoting monounsaturated fat intake. In addition, the presence of dietary fiber, minimal sugar, and low moisture content enhance its functionality in shelf-stable and health-oriented formulations. HOAP’s natural matrix eliminates the need for emulsifiers or complex encapsulation systems, reducing formulation complexity and improving compatibility with clean-label product strategies.

In addition to its compositional and stability advantages, HOAP exhibits functional properties that are highly relevant for food processing applications. As a naturally structured biomass with intracellular lipid droplets embedded in a protein–fiber matrix, HOAP differs fundamentally from conventional oil powders that rely on emulsifier-based encapsulation systems [1]. When conventional powdered oils are added to a water continuous phase, the shell usually dissolves, releasing the oil. When added to an oil continuous phase with some shear, the oil dissolves leaving the power dispersed in the oil. HOAP can be added to water or oil continuous systems with shear creating a dispersion, but the oil remains inside the cell. The oil in HOAP is not externally emulsified but instead retained within the intact cellular structure of the microalgae, providing both oxidative protection and processing flexibility. However, to fully access the lipid fraction for formulation purposes, the mechanical disruption of the matrix is often required. Several processing techniques can be employed to facilitate oil release and integration from HOAP into food matrices. High-pressure homogenization (HPH) at pressures up to 1500 bar has proven effective in disrupting the microalgal cell walls, enabling the release of intracellular oil into homogenous emulsions [23,24]. This approach is particularly well-suited for applications such as plant-based beverages, infant nutrition, or clinical nutrition formulas, where lipid dispersion and emulsion stability are critical. Bead milling is another effective method for unlocking HOAP’s lipid content. This high-shear mechanical process breaks down the cell wall and disperses the oil within an aqueous phase, yielding a smooth lipid-rich paste. As shown in Supplementary Figure S2, the conversion from the HOAP to a homogenous paste can be achieved by milling the HOAP. This processing approach is advantageous for applications such as culinary sauces, dairy analogs, or functional spreads. Additionally, HOAP can be incorporated into dry and semi-moist applications through extrusion. The high temperature, pressure, and shear forces encountered during extrusion can be sufficient to disrupt the microalgal matrix, resulting in oil release during processing. This makes HOAP suitable for use in baked goods, extruded snacks, and plant-based meat alternatives, where it can serve both as a lipid source and as a structuring agent.

Furthermore, the robust stability profile under both ambient and elevated temperature conditions supports its use in a wide range of applications, including nutritional bars, plant-based dairy and meat analogues, powdered meal replacements, and bakery products. The combined nutritional, functional, and stability attributes of HOAP offer a differentiated value proposition relative to traditional oil powders, expanding formulation possibilities while aligning with consumer demand for minimally processed and nutritionally beneficial ingredients.

## 5. Conclusions

This study shows that HOAP, derived from *Prototheca moriformis*, is a stable, lipid-rich, clean-label ingredient with high oleic acid and fiber, low sugar and moisture, and a native dry format. Future work should validate its functionality in foods, its role as a bioactive carrier, and its fit as a structured fat to meet consumer and industry needs.

## 6. Patents

The strain used to produce the HOAP described in the present paper is described in the patent US011873405B entitled “High-oleic oil compositions and uses thereof”.

## Figures and Tables

**Table 1:** Key performance indicators (KPIs) for high oleic strain in 20 L fermenters.

Fermentation Run	Oleic Acid (% of Total FA)	Dried-Cell Weight (DCW, g per kg)	Oil Titer (g per L)	Oil Content (% of DCW)
Batch 1	88.8	212.0	129.3	61.0
Batch 2	88.8	210.0	128.1	61.0
Batch 3	89.0	208.0	133.1	64.0
Average	88.9	210.0	130.2	62.0
Standard deviations	0.1	2.0	2.6	1.7
Coefficient of variation (%;)	0.1	1.0	2.0	2.8

**Table 2:** Proximate analysis of 3 production batches of high-oleic algal powder (HOAP). Data are expressed in g per 100 g of HOAP. SD and CV% stands for standard deviation and coefficient of variation expressed in %.

	Batch 1	Batch 2	Batch 3	Average	SD	CV%
Moisture	1.6	2.8	2.8	2.4	0.7	28.9
Fiber	33.1	37.9	32.1	34.4	3.1	9.0
Ash	1.3	1.2	1.3	1.3	0.1	4.6
Protein	3.2	3.3	3.1	3.2	0.1	3.1
Fat	63.1	65.3	65.2	64.5	1.2	1.9
Sucrose	1.3	1.2	1.4	1.3	0.1	7.7

**Table 3:** Fatty acid composition of 3 production batches of high-oleic algal powder (HOAP). Data are expressed in % of total fatty acids. SD and CV% stands for standard deviation and coefficient of variation expressed in %.

	Batch 1	Batch 2	Batch 3	Average	SD	CV%
14:0	0.22	0.23	0.23	0.23	0.01	2.55
16:0	3.67	3.78	3.71	3.72	0.06	1.50
16:1 n–7	0.16	0.18	0.17	0.17	0.01	5.88
18:0	1.76	1.62	1.76	1.71	0.08	4.72
18:1 n–9	88.83	88.77	89.04	88.88	0.14	0.16
18:2 n–6	3.31	3.49	3.19	3.33	0.15	4.53
18:3 n–3	0.24	0.25	0.24	0.24	0.01	2.37
20:0	0.22	0.19	0.21	0.21	0.02	7.39
20:1 n–9	1.05	1.00	1.04	1.03	0.03	2.57
Other fatty acids	0.56	0.51	0.43	0.50	0.07	13.11

**Table 4:** Amino acid composition of 3 production batches of high-oleic algal powder (HOAP). Data are expressed in g per 100 g of HOAP. SD and CV% stands for standard deviation and coefficient of variation expressed in %.

	Batch 1	Batch 2	Batch 3	Average	SD	CV%
Glutamic acid	0.29	0.34	0.35	0.33	0.03	9.84
Alanine	0.28	0.26	0.26	0.27	0.01	4.33
Aspartic acid	0.27	0.26	0.24	0.26	0.02	5.95
Leucine	0.27	0.27	0.25	0.26	0.01	4.38
Valine	0.21	0.19	0.19	0.20	0.01	5.87
Arginine	0.2	0.18	0.16	0.18	0.02	11.11
Lysine	0.18	0.17	0.16	0.17	0.01	5.88
Serine	0.16	0.15	0.18	0.16	0.02	9.35
Glycine	0.17	0.16	0.16	0.16	0.01	3.53
Proline	0.18	0.14	0.14	0.15	0.02	15.06
Threonine	0.15	0.15	0.16	0.15	0.01	3.77
Phenylalanine	0.15	0.13	0.14	0.14	0.01	7.14
Isoleucine	0.12	0.12	0.11	0.12	0.01	4.95
Tyrosine	0.1	0.09	0.1	0.10	0.01	5.97
Histidine	0.06	0.07	0.07	0.07	0.01	8.66
Cysteine + cystine	0.05	0.06	0.06	0.06	0.01	10.19
Tryptophan	0.05	0.05	0.05	0.05	0.00	0.00
Methionine	<0.02	0.06	0.05	0.06	0.01	12.86
Hydroxyproline	<0.20	<0.20	<0.20	<0.20	-	-
Ornithine	<0.05	<0.05	<0.05	<0.05	-	-

**Table 5:** Biogenic amine analysis of 3 production batches of high-oleic algal powder (HOAP). Data are expressed in mg per kg of HOAP.

	Batch 1	Batch 2	Batch 3	Average	SD	CV%
Cadaverine	<1	<1	<1	<1	-	-
Histamine	<1	<1	<1	<1	-	-
Phenylethylamine	<1	<1	<1	<1	-	-
Putrescine	<1	<1	<1	<1	-	-
Spermidine	68.0	76.8	79.5	74.8	6.0	8.0
Spermine	<1	<1	<1	<1	-	-
Tryptamine	<5	<5	<5	<5	-	-
Tyramine	<1	<1	<1	<1	-	-

**Table 6:** Results of the stability test conducted for 2 months of high-oleic algal powder (HOAP) at 23 and 45 °C with or without addition of BHT.

Parameter	Temperature	Samples	Time		
			0 Months	1 Month	2 Months
Peroxide Value (meq O_2_/kg)	23 °C	0 ppm	0.9	1.4	2.8
		20 ppm	0.8	0.9	0.5
		100 ppm	0.5	1.5	0.6
	45 °C	0 ppm	0.9	3.5	3.5
		20 ppm	0.8	2.1	2.3
		100 ppm	0.5	2.3	2.3
Fat Content (g/100 g)	23 °C	0 ppm	58.65	60.03	59.72
		20 ppm	60.19	59.27	59.64
		100 ppm	58.29	59.18	59.17
	45 °C	0 ppm	58.65	60.13	59.85
		20 ppm	60.19	60.46	61.21
		100 ppm	58.29	59.94	59.66
Appearance	23 °C	0 ppm	Conforms *	Conforms	Conforms
		20 ppm	Conforms	Conforms	Conforms
		100 ppm	Conforms	Conforms	Conforms
	45 °C	0 ppm	Conforms	Conforms	Conforms
		20 ppm	Conforms	Conforms	Conforms
		100 ppm	Conforms	Conforms	Conforms

* Conforms refer to a pale-yellow powder appearance as displayed in Figure 1.

**Table 7:** Microbial data of 3 production batches of high-oleic algal powder (HOAP). Data are expressed as CFU per gram of HOAP.

	Batch 1	Batch 2	Batch 3
Aerobic Plate Count	3000	<1000	80
Coliforms	<10	<10	<10
*E. coli*	<10	<10	<10
*Salmonella*	Not detected *	Not detected *	Not detected *
Coagulase-positive *Staphylococci*	<10	<10	<10
*Pseudomonas aeruginosa*	Not detected	Not detected	Not detected
Mesophilic aerobic spores	<10	<10	<10
Mold + yeast	<1000	<1000	<1000
Mold	<1000	<1000	<1000
Yeast	<1000	<1000	<1000

* Not detected in 25 g.

## Data Availability

The original contributions presented in the study are included in the article/Supplementary Materials; further inquiries can be directed to the corresponding author.

## Supplementary Materials

The following supporting information can be downloaded at https://www.mdpi.com/article/10.3390/microorganisms13071659/s1, Table S1: BLASTp results and annotations for hits between *P. moriformis* base strain and COMPARE allergen genes. The first two columns (query and annotation) describe *P. moriformis* proteins. The next six columns (allergen match, percent ID, match length, mismatch, E-value, Bit score) describe components of BLAST similarity. The next three columns (genus/species, common name, description) describe the allergen matches. The final two columns (IgE match and IgE motif) describe follow-up searches of IgE databases; Figure S1: Venn diagram of protein coding genes from *Prototheca moriformis* base strain, Auxenochlorella, and Chlorella that matched COMPARE database allergen genes; Figure S2: Appearance of the high-oleic algal powder ingredient before and after bead milling.

## Abbreviations

The following abbreviations are used in this manuscript:

4-NQO	4-Nitroquinoline 1-Oxide
AOAC	Association of Official Agricultural Chemists
ATCC	American Type Culture Collection
BHT	Butylated Hydroxytoluene
BLAST	Basic Local Alignment Search Tool
BSL-1	Biosafety Level 1
DCW	Dry Cell Weight
DO	Dissolved Oxygen
EMS	Ethylmethane Sulfonate
FA	Fatty Acid
FAMEs	Fatty Acid Methyl Esters
HOAP	High-Oleic Algal Powder
HPH	High-Pressure Homogenization
IgE	Immunoglobulin E
ISO	International Organization for Standardization
NF	Norme Française (French Standard)
UTEX	University of Texas Culture Collection of Algae
