# Supplementary material for: Fermentation of Microalgae as a Platform for Naturally Encapsulated Oil Powders: Characterization of a High-Oleic Algal Powder Ingredient
Source: Microorganisms. 2025 Jul 14;13(7):1659. doi: 10.3390/microorganisms13071659 (PMC12298329; doi:10.3390/microorganisms13071659)
Supplement: Supplementary file 1 [file microorganisms-13-01659-s001.zip › Supplementary Table S1.pdf]

SUPPLEMENTARY MATERIAL

Table S1. Blastp results and annotations for hits between *P. moriformis* base strain and COMPARE allergen genes. The first two columns (query and annotation) describe *P. moriformis* proteins. The next six columns (allergen match, percent ID, match length, mismatch, E-value, Bit score) describe components of BLAST similarity. The next three columns (genus/species, common name, description) describe the allergen matches. The final two columns (IgE match and IgE motif) describe follow up searches of IgE databases.

Query	Annotation	Allergen Match	Percent ID	Match Length	Mismatch	E-value	Bit Score	Species	Common Name	Description	IgE Match	IgE Motif
g1401	Tubulin alpha chain	AAX84656.1	83.111	450	76	0	799	*Tyrophagus putrescentiae*	Storage mite	Alpha-tubulin	No	No
g3799	Casein kinase II subunit alpha-1	XP)015646887.1	77.108	332	76	0	506	*Oryza sativa Japonica Group*	Japanese Rice	PREDICTED: casein kinase II subunit alpha	No	No
g343	None	sp P40918.1 HSP70_DAVTA	72.903	620	161	0	929	*Cladosporium herbarum*	Fungus	Heat shock 70 kDa protein (Allergen Cla h 4) (Cla h IV)	No	No
g17	Glyceraldehyde-3-phosphate dehydrogenase	CAZ76054.1	72.754	334	88	4.32E-172	477	*Triticum aestivum*	Wheat	Glyceraldehyde-3-phosphate-dehydrogenase	No	No
g2102	Peptidyl-prolyl cis-trans isomerase CYP19-4	AEY79726.1	72.455	167	46	9.81E-90	257	*Daucus carota*	Carrot	Cyclophilin [Daucus carota]	No	No
g3786	Peptidyl-prolyl cis-trans isomerase CYP18-4	sp P81531.2 CYPH_BETPN	70.455	44	11	7.04E-14	58.2	*Betula pendula*	European white birch	Cyclophilin	No	No
