# Supplementary figures and images for: Fermentation of Microalgae as a Platform for Naturally Encapsulated Oil Powders: Characterization of a High-Oleic Algal Powder Ingredient
Source: Microorganisms. 2025 Jul 14;13(7):1659. doi: 10.3390/microorganisms13071659 (PMC12298329; doi:10.3390/microorganisms13071659)

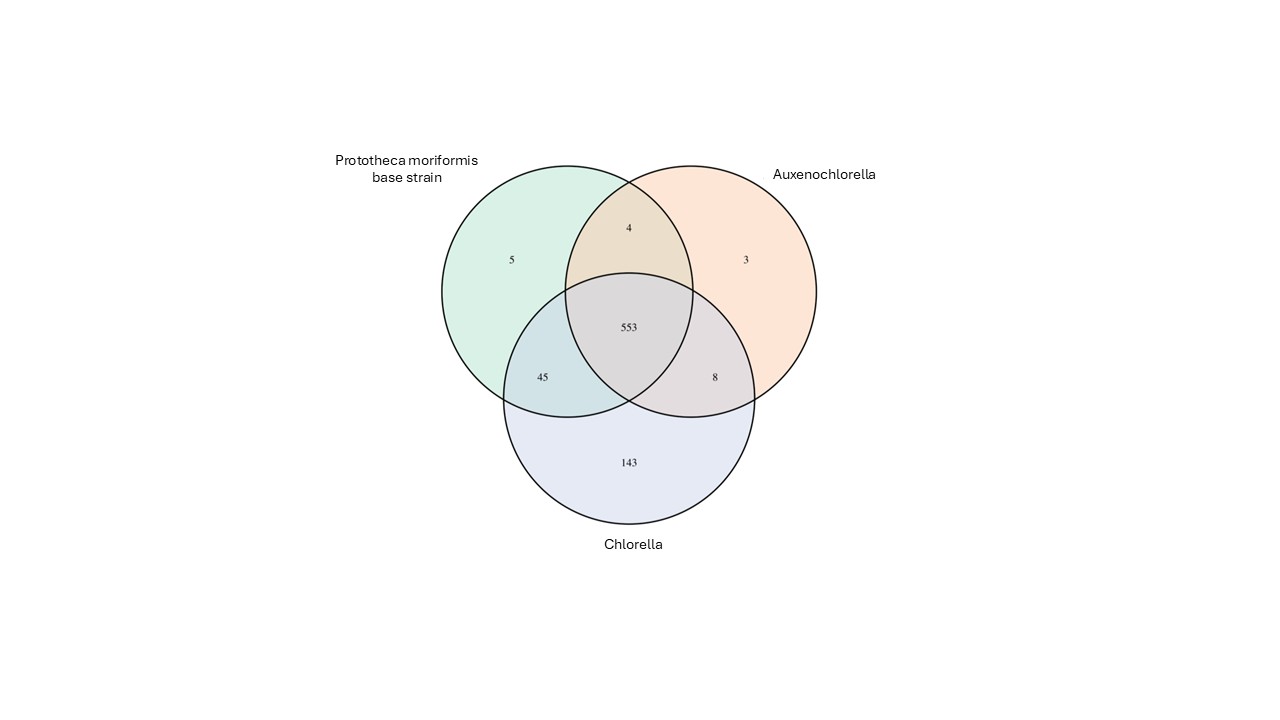

Supplement: Supplementary file 1 [file microorganisms-13-01659-s001.zip › Suppl Figure S1.jpg]

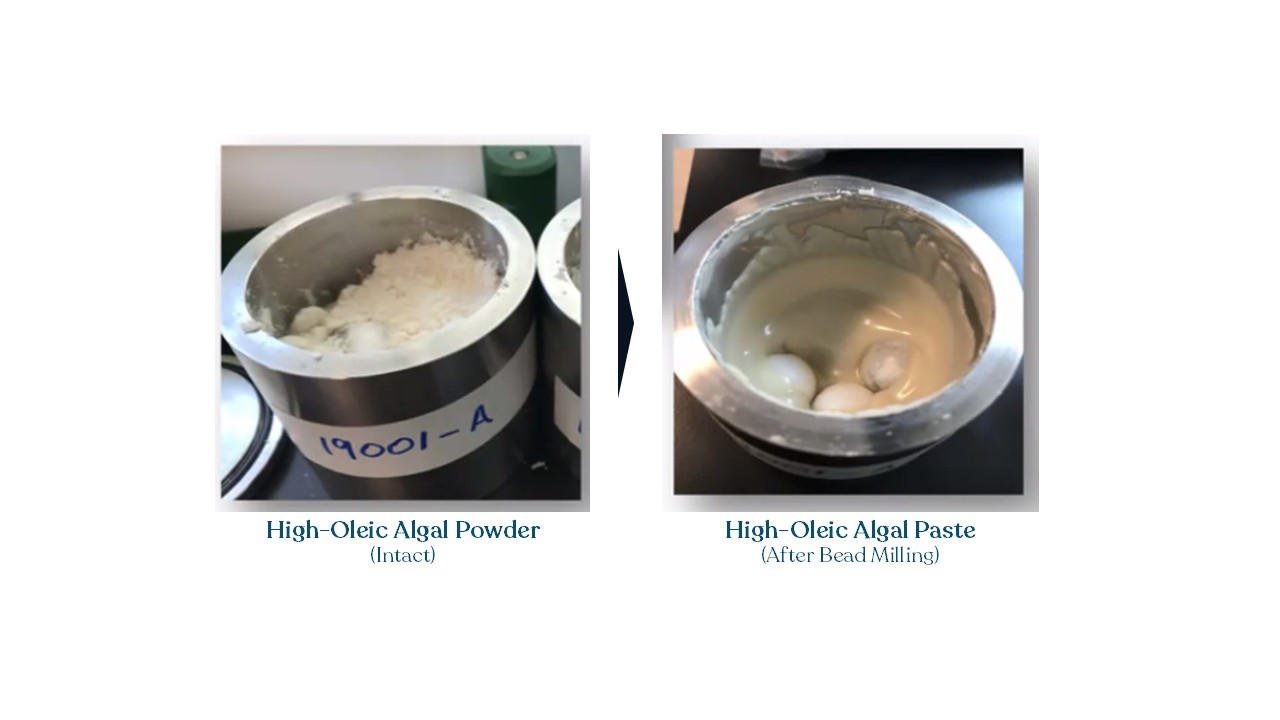

Supplement: Supplementary file 1 [file microorganisms-13-01659-s001.zip › Suppl Figure S2.jpg]
